# A novel phage preparation capable of simultaneously inhibiting both K1 and K2 serotypes of *Klebsiella pneumoniae*

**DOI:** 10.1128/aem.02423-25

**Published:** 2026-02-13

**Authors:** Zhibin Zhang, Na Ling, Xinran Li, Diwei Zhang, Zhixiang Nie, Shengshi Jiang, Yingwang Ye

**Affiliations:** 1 School of Food Science and Engineering, Hefei University of Technology 558979, Hefei, China; 2 School of Food and Biological Engineering, Key Laboratory for Animal Food Green Manufacturing and Resource Mining of Anhui Province, Engineering Research Center of Bio-Process, Ministry of Education, Hefei University of Technology 558979, Hefei, China; 3 National Health Commission Science and Technology Innovation Platform for Nutrition and Safety of Microbial Food, Guangdong Provincial Key Laboratory of Microbial Safety and Health, State Key Laboratory of Applied Microbiology Southern China, Institute of Microbiology, Guangdong Academy of Sciences, Guangzhou, China; University of Nebraska-Lincoln, Lincoln, Nebraska, USA

**Keywords:** *Klebsiella pneumoniae *phage, production optimization, RSM, expanded production

## Abstract

Given the high pathogenicity and escalating antimicrobial resistance of *Klebsiella pneumoniae*, the efficacy of conventional antibiotics is waning, creating an urgent need for alternative therapeutic strategies. Phage therapy has emerged as a particularly promising approach for combating highly virulent and multidrug-resistant *K. pneumoniae* due to its target specificity and potent bactericidal activity. This study isolated a lytic *K. pneumoniae* phage vB_kpnP_ZB27 (ZB27) from domestic sewage in Hefei, China. This phage demonstrated specificity against clinically relevant serotypes K1 and K2. It exhibited remarkable stability across a wide range of temperatures (20°C–50°C) and pH values (4–11). Crucially, genomic analysis confirmed the absence of antibiotic resistance or virulence genes. These findings collectively indicated that phage ZB27 is a promising and safe candidate for phage therapy. The core objective of this study was to optimize the phage fermentation process. To enhance phage yield, we optimized inoculum concentration and medium composition. Laboratory-scale fermentation determined the optimal multiplicity of infection to be 0.0001. Furthermore, response surface methodology optimized key additive concentrations: sorbitol to 46.8 mg/mL, soybean meal to 59.9 mg/mL, and calcium chloride to 1 mM. This optimized protocol increased phage yield by 2.96-fold during scale-up, establishing an effective method for industrial production of therapeutic phages.

**IMPORTANCE:**

Traditional phage preparations typically consist of a single phage with a narrow host range, often requiring the use of complex phage cocktails to cover target strains, which substantially increases production costs. In this study, we successfully isolated broad-spectrum phages capable of lysing both K1 and K2 serotypes, thereby significantly expanding the bactericidal spectrum of the phage preparation. Furthermore, through industrial process optimization, we achieved dual benefits: reducing host bacterial consumption while increasing phage yield. These findings demonstrate that artificially optimized production processes can improve both the economic feasibility and safety of phage biomanufacturing, thus opening new pathways for the industrialization of phage therapy.

## INTRODUCTION

*Klebsiella pneumoniae*, as a notorious pathogenic microorganism, poses a serious threat to global health safety. It can cause a series of clinical diseases such as meningitis, bacteremia, and liver abscesses in infected individuals (1, 2). Hypervirulent *K. pneumoniae* (hvKP), notably serotypes K1 and K2, pose a severe threat due to their tendency to metastasize to sites such as the eyes and the central nervous system (3). In contrast to classical *K. pneumoniae*, which typically infects immunocompromised individuals, these hvKP strains are capable of infecting healthy hosts and have already developed antimicrobial resistance (4, 5). It is reported that the global prevalence rates of carbapenem-resistant hypervirulent *K. pneumoniae* exceed 40% (6). In addition to clinical manifestations, *K. pneumoniae* also exhibits wide distribution in soil, water resources, and other environments (7–10), revealing the potential threat of *K. pneumoniae* to agriculture or food. Recently, detection of carbapenem-resistant *K. pneumoniae* in orchard soil has heightened concerns about agricultural food security (11). Consequently, the development of new biological control is of crucial significance to guarantee agricultural food security and create a green and hygienic environment.

Phages have become favorable antimicrobial agents against *K. pneumoniae*, especially drug-resistant strains, due to their safety, efficiency, and specificity (12). Numerous studies have emphasized the clinical potential of phage therapy through some animal experiments (13–16). However, phage doses used for clinical animal models are typically very low. The practical application of phage therapy requires efficient large-scale production methods, especially the optimization of phage fermentation processes (17–20). Lytic phages cannot be produced without host bacteria, which means that the intensive interaction between the phage and the host bacterium will benefit the growth of the phage (21). Meanwhile, some research has also shown that condition factors such as temperature and nutrients play a key role in phage growth (22, 23). This indicates the possibility of increasing the final yield of phage by optimizing the phage fermentation conditions.

This study isolated ZB27, a highly efficient lytic phage capable of targeting K1 and K2 strains, from sewage. It was found to exhibit excellent tolerance to temperature and pH variations, and genomic analysis revealed that ZB27 lacks virulence and antibiotic resistance genes. In addition, we optimized the phage production by adjusting the culture conditions during phage fermentation, such as changing the phage inoculum concentration, setting different multiplicities of infection (MOIs), and supplementing different growth medium additives. We also analyzed the results using response surface methodology (RSM) to optimize the fermentation combinations.

## MATERIALS AND METHODS

### Experimental strain and phage

A K1-type *K. pneumoniae* strain, Kp 202, was used in this study. LB broth and agar media were used for bacterial culture and phage proliferation. Kp 202 was stored at −80°C in LB broth and supplemented with 20% glycerol. To obtain a bacterial suspension, the strain was streaked onto an LB agar plate using an inoculation loop, and one single colony was then transferred to LB broth and incubated overnight at 37°C with shaking (200 rpm). Following a previously reported method (24) with minor modifications, a *K. pneumoniae* phage strain, ZB27, was isolated from wastewater. For high-concentration phage preparation, overnight-cultured Kp 202 was co-inoculated with phage ZB27 into fresh LB broth and incubated overnight at 37°C with shaking (200 rpm). The culture was centrifuged (8,000 × g), the supernatant was filtered (0.45 μm), and phage particles were concentrated by PEG 8000 precipitation. The resulting high-titer phage solution was stored at 4°C.

### Phage proliferation and titer determination

For phage propagation, the method was the same as described above. The phage titer was determined using the double agar plate method. Briefly, 100 μL of the logarithmic-phase host bacterial fluid and the gradient-diluted phage proliferation solution were mixed in 5 mL of 0.4% LB top agar, then poured onto a 1.5% bottom agar plate. After overnight incubation at 37°C, the plaque-forming units (PFUs) can be determined.

### Host range assay

The host range was determined using a spot assay. Briefly, a base layer of LB agar (1.5%) was prepared and allowed to solidify in sterile Petri dishes. Then, 100 μL of logarithmic-phase bacterial cultures from various strains were mixed with 5 mL of soft LB agar (0.4%) and overlaid onto the base agar. After the overlay solidified, 5 μL of phage lysate was applied as spots onto the surface. The plates were incubated overnight at 37°C and examined for plaque formation.

### One-step growth assay

To determine the one-step growth curve of phage vB_kpnP_ZB27, a slightly modified version of a previously described method was employed (25). Briefly, the logarithmic-phase host bacteria were infected with the phage at the optimal MOI and incubated at 37°C for 5 min. The mixture was then centrifuged at 12,000 × *g* for 30 s to remove unadsorbed phages. The pellet was washed twice with LB broth and resuspended in 30 mL of fresh medium. The culture was incubated at 37°C with shaking at 200 rpm. Samples were collected every 10 min, immediately centrifuged (12,000 × *g*, 30 s), and filtered through a 0.45 μm membrane. The filtrates were titrated by plaque assay, and the one-step growth curve was plotted with the incubation time on the *x*-axis and the phage titer on the *y*-axis.

### Antibacterial activity assay

An experiment was designed to assess the antibacterial activity of phage ZB27. The initial bacterial concentration was adjusted to 1 × 10⁸ (OD_600_ ≈ 0.6), and phage was added at varying ratios. The mixture was incubated at 37°C with shaking at 200 rpm, with OD_600_ measurements taken every hour. The bacteriostatic activity of phage ZB27 was determined by assessing the turbidity of the culture.

### Thermal and pH stability

The environmental stability of phage ZB27, including its tolerance to temperature and pH, was evaluated (26). For thermal stability, the phage was incubated at temperatures ranging from 20 to 70°C (in 10°C increments) for 1 h. For pH stability, 100 μL of the phage suspension was mixed with 900 μL of LB broth adjusted to different pH values (2–13) and incubated at 37°C for 1 h (13). Following each treatment, the phage titer was quantified using the double-layer agar plaque assay.

### Phage genome DNA extraction, sequencing, and genome analysis

The DNA of phage ZB27 was extracted using a viral genome extraction kit (Solarbio, Beijing, China) and subsequently subjected to whole-genome sequencing via Illumina technology. Qualified libraries were sequenced on the Illumina HiSeq X Ten platform (Illumina, USA) using 150 bp paired-end reads. Read QC was performed using Cutadapt 1.9.1 software, and assembly was completed with Velvet 1.2.10 software. Gap filling was achieved using SSPACE 3.0 and GapFiller 1-10 software, respectively. Whole-genome sequence alignment was conducted against the NCBI database (https://static.pubmed.gov/portal/portal.fcgi/). We retrieved the 30 phage genomes most closely related to ZB27 from NCBI and generated a similarity heatmap using VIRIDIC (27). For phage protein annotation, analyses were performed with Blastp https://static.pubmed.gov/portal/portal.fcgi/., HHpred (https://toolkit.tuebingen.mpg.de/) (28), and the RCSB PDB database (https://www.rcsb.org/). The genomic map of phage ZB27 was generated using Circular Genome Viewer (CGView) (29). Complete phage genomes were visualized with Easyfig 2.2.5 (30). To assess potential lysogenic or pathogenic traits, predicted virulence factors and antibiotic resistance genes were checked against the Virulence Factors Database and the Antibiotic Resistance Database, respectively. Additional genomic sequences of related phages were obtained from the Virus-Host Database.

For phylogenetic reconstruction, a proteome-based tree was generated with the ViPTree server and visualized in iTOL (v.7.2.2) (https://itol.embl.de/) (31). To further evaluate evolutionary relationships and taxonomic novelty, a maximum-likelihood phylogenetic tree based on major capsid proteins was constructed using MEGA 11 with 1,000 bootstrap replicates, and the resulting tree was also visualized in iTOL.

### Effect of MOI on phage fermentation

To evaluate the impact of the MOI on phage production, infections were carried out at different MOIs (1, 0.1, 0.01, 0.001, 0.0001, and 0.00001). The initial concentration of the host bacteria was standardized to 1 × 10⁸ CFU/mL. The phage concentration was then adjusted accordingly to achieve the target MOI. A mixture containing 100 μL of the bacterial suspension, 100 μL of the phage lysate, and 5 mL of fresh LB broth was incubated at 37°C. The phage titer was determined by plaque assay when the culture turbidity became evident.

### Effect of different culture medium additives on phage fermentation

To better investigate the effect of culture medium supplements on phage production, LB broth was replaced with Nutrient Broth, which has a simpler formulation. The host bacteria were inoculated into 50 mL of NB medium and cultured to the exponential phase, then the phage was inoculated at the optimal MOI. In the carbon source optimization experiment, 0.5% (wt/vol) soluble starch, glucose, glycerol, sorbitol, and sucrose were supplemented to the NB. For the nitrogen source, 0.3% (wt/vol) ammonium sulfate, soybean meal, peanut cake meal, soybean peptone, and yeast powder were added to examine the effect of the nitrogen source on phage production. We also selected magnesium chloride and calcium chloride to evaluate the effect of divalent metal cations on phage production; their concentration was set to 1 mM. All experiments were conducted in 100 mL flasks and analyzed using the one-factor-at-a-time method.

### Experimental design for optimization of fermentation conditions

The experimental design employed the RSM based on the Box-Behnken Design (BBD) with the objective of ascertaining the optimal levels of the selected variables (carbon source, nitrogen source, and metal ions). The design was implemented using Design Expert software (v.13.0.15), generating a total of 17 different coding levels. To ensure the robustness of the findings, three replicate experiments were conducted for each coding level. Finally, the relationship between phage production and the level of each factor was modeled using the following binomial statistics:


$$P=a_{0}+\sum a_{i}X_{i}+\sum a_{ij}X_{i}X_{j}+\sum a_{ii}X_{i}^{2}$$

where *P* represents the predicted phage yield, *a*_0_ is the constant term, *a*_*i*_ is the linear coefficient, *a*_*ii*_ is the quadratic coefficient, and *a*_*ij*_ is the interaction coefficient. *X*_*i*_ is the independent variable, *X*_*i*_^2^ is the independent variable squared term, and *X*_ij_ is the interaction between the independent variables. Response surfaces were generated using Design Expert (v.13.0.15) for visualization. Analysis of variance (ANOVA) was used to assess the validity of the model, which indicated statistical significance if *P* < 0.05. 

### Phage validation at fermenter scale

After RSM optimization of the fermentation process of phage ZB27, the feasibility of large-scale production of ZB27 in fermenters was evaluated. The fermentation process was carried out in a 50 L fully automated fermentation system with temperature and rotational speed conforming to laboratory standards. No additives were added to the control group. Similarly, the fermentation liquid was collected after 8 h of incubation, and titers were measured by double-layer agar method.

### Statistical analysis

All measurements were carried out in triplicate unless otherwise specified, and the data are reported as the mean ± standard deviation. Statistical differences were calculated by ordinary one-way ANOVA with multiple comparisons.

## RESULTS

### Phage isolation and morphology

**Fig 1:**
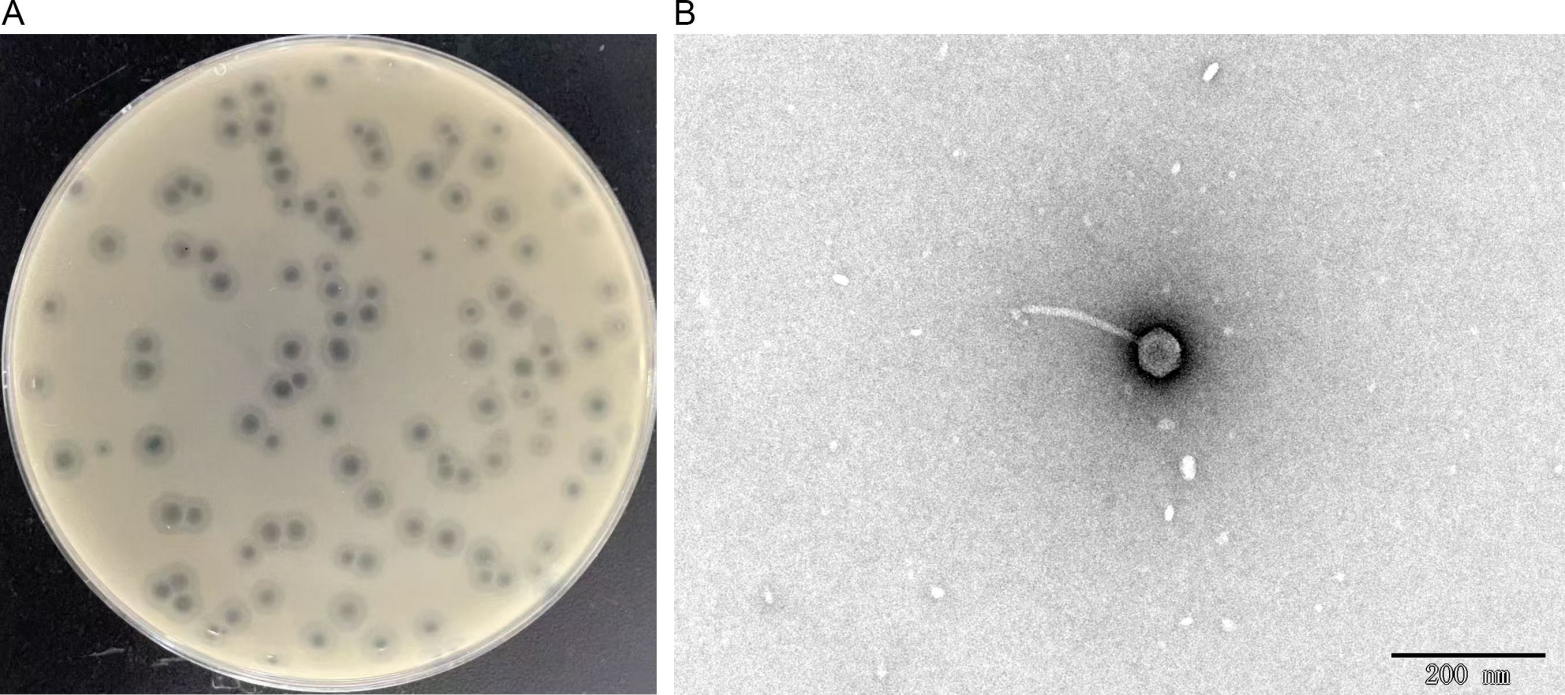
Phage ZB27 morphology. (**A**) Phage plaque formed on a double-layer agar plate. (**B**) Transmission electron micrograph of ZB27. Scale bar: 200 nm.

The *K. pneumoniae* phage vB_kpnP_ZB27 was isolated from a wastewater treatment plant in Hefei and is deposited in the China Center for Type Culture Collection (CCTCC NO: M 20252087). On a double-layer agar plate, it forms distinct plaques with clear, transparent halos approximately 2 mm in diameter, suggesting potent lytic activity (Fig. 1).

### Host range

Forty-five strains of different *K. pneumoniae* types were used to determine the host range of phage ZB27. Results indicated that phage ZB27 could lyse only 11 *K. pneumoniae* strains of two K types (7 K1 strains and 4 K2 strains) (Table 1).

**TABLE 1:** Host range of phage ZB27^*a*^

Species	Strain	Serotype	Lysis	Source
*K. pneumoniae*	Kp176	K1	+	Laboratory collection
*K. pneumoniae*	Kp177	K2	+	Laboratory collection
*K. pneumoniae*	Kp178	K1	+	Laboratory collection
*K. pneumoniae*	Kp179	K64	−	Laboratory collection
*K. pneumoniae*	Kp180	K1	+	Laboratory collection
*K. pneumoniae*	Kp181	K20	−	Laboratory collection
*K. pneumoniae*	Kp182	NT	−	Laboratory collection
*K. pneumoniae*	Kp183	K30	−	Laboratory collection
*K. pneumoniae*	Kp184	K5	−	Laboratory collection
*K. pneumoniae*	Kp185	K1	+	Laboratory collection
*K. pneumoniae*	Kp186	K1	+	Laboratory collection
*K. pneumoniae*	Kp187	K20	−	Laboratory collection
*K. pneumoniae*	Kp188	K2	+	Laboratory collection
*K. pneumoniae*	Kp189	K64	−	Laboratory collection
*K. pneumoniae*	Kp190	K35	−	Laboratory collection
*K. pneumoniae*	Kp191	K35	−	Laboratory collection
*K. pneumoniae*	Kp192	K35	−	Laboratory collection
*K. pneumoniae*	Kp193	K54	−	Laboratory collection
*K. pneumoniae*	Kp194	K54	−	Laboratory collection
*K. pneumoniae*	Kp195	K57	−	Laboratory collection
*K. pneumoniae*	Kp196	K50	−	Laboratory collection
*K. pneumoniae*	Kp197	K35	−	Laboratory collection
*K. pneumoniae*	Kp198	K57	−	Laboratory collection
*K. pneumoniae*	Kp199	K54	−	Laboratory collection
*K. pneumoniae*	Kp200	K5	−	Laboratory collection
*K. pneumoniae*	Kp201	K31	−	Laboratory collection
*K. pneumoniae*	Kp202	K1	+	Laboratory collection
*K. pneumoniae*	Kp203	K57	−	Laboratory collection
*K. pneumoniae*	Kp204	K80	−	Laboratory collection
*K. pneumoniae*	Kp205	K1	+	Laboratory collection
*K. pneumoniae*	Kp206	NT	−	Laboratory collection
*K. pneumoniae*	Kp207	K2	+	Laboratory collection
*K. pneumoniae*	Kp208	K27	−	Laboratory collection
*K. pneumoniae*	Kp209	K27	−	Laboratory collection
*K. pneumoniae*	Kp210	NT	−	Laboratory collection
*K. pneumoniae*	Kp211	K24	−	Laboratory collection
*K. pneumoniae*	Kp212	NT	−	Laboratory collection
*K. pneumoniae*	Kp213	K38	−	Laboratory collection
*K. pneumoniae*	Kp214	K57	−	Laboratory collection
*K. pneumoniae*	Kp215	K64	−	Laboratory collection
*K. pneumoniae*	Kp216	K2	+	Laboratory collection
*K. pneumoniae*	Kp217	K1	+	Laboratory collection
*K. pneumoniae*	Kp218	K26	−	Laboratory collection
*K. pneumoniae*	Kp219	K5	−	Laboratory collection
*K. pneumoniae*	Kp220	NT	−	Laboratory collection

^*a*^ All 45 *Klebsiella pneumoniae* strains used in this experiment were obtained from the laboratory collection. +, phage sensitivity to the strain; −, phage are not sensitive to strains.

### Phage biological characteristics

**Fig 2:**
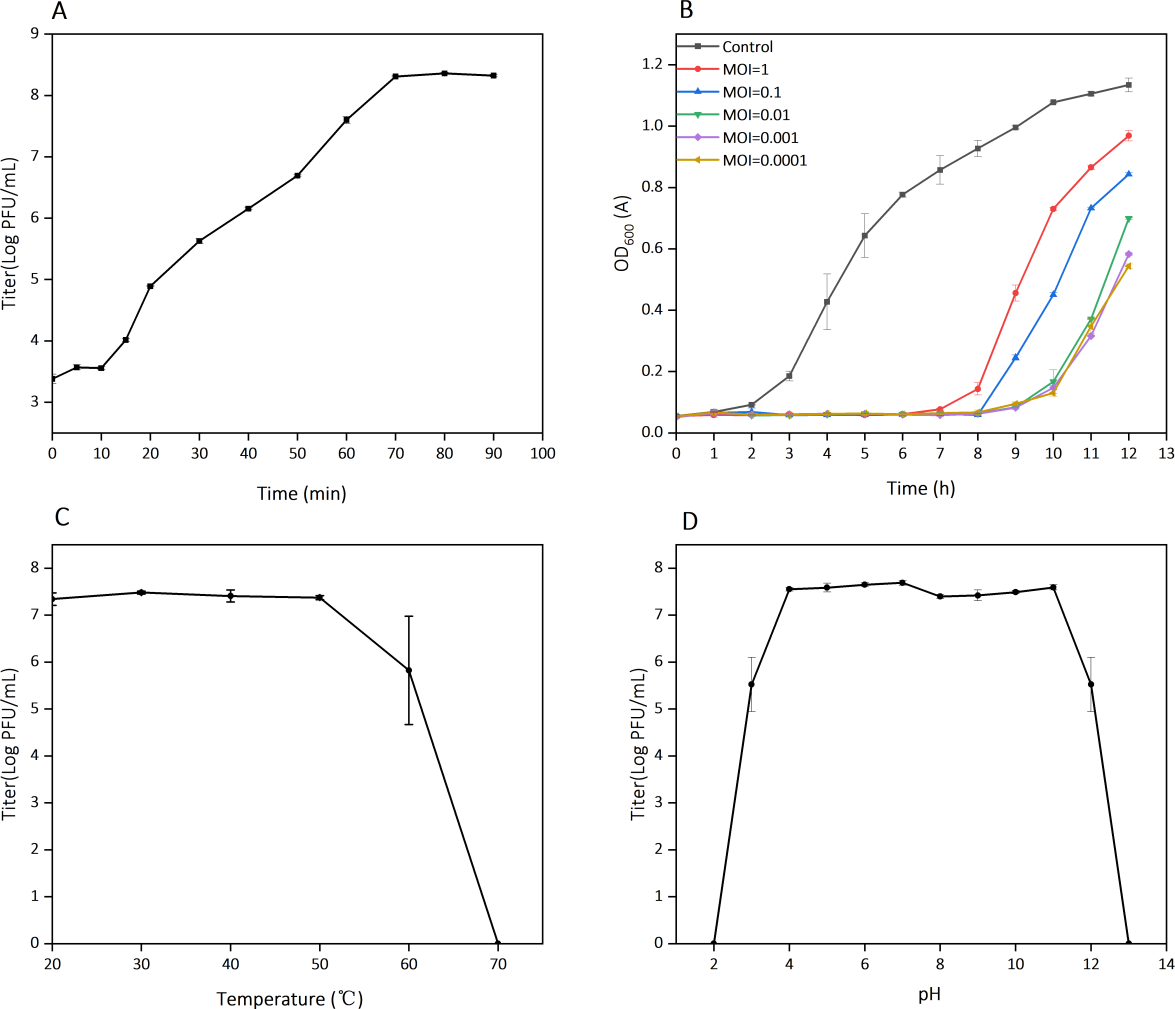
Characteristics of phage ZB27. (**A**) One-step growth curve. (**B**) Antibacterial activity. (**C**) Temperature stability. (**D**) pH stability. Three parallel experiments were performed.

The replication cycle of phage ZB27 was characterized using a one-step growth curve, which revealed a 10 min latent period, a 60 min exponential phase, and a plateau phase commencing at 70 min (Fig. 2A). ZB27 demonstrates potent antibacterial activity, significantly inhibiting bacterial growth over an 8 h period at various MOIs (Fig. 2B). Moreover, the phage exhibits remarkable stability across a broad temperature range (20°C–50°C) and pH values (4–11), with no significant titer reduction observed under these conditions (Fig. 2C and D). This robust stability underscores the potential of phage ZB27 as a highly suitable candidate for use as an effective antibacterial agent.

### Phage genome analysis

**Fig 3:**
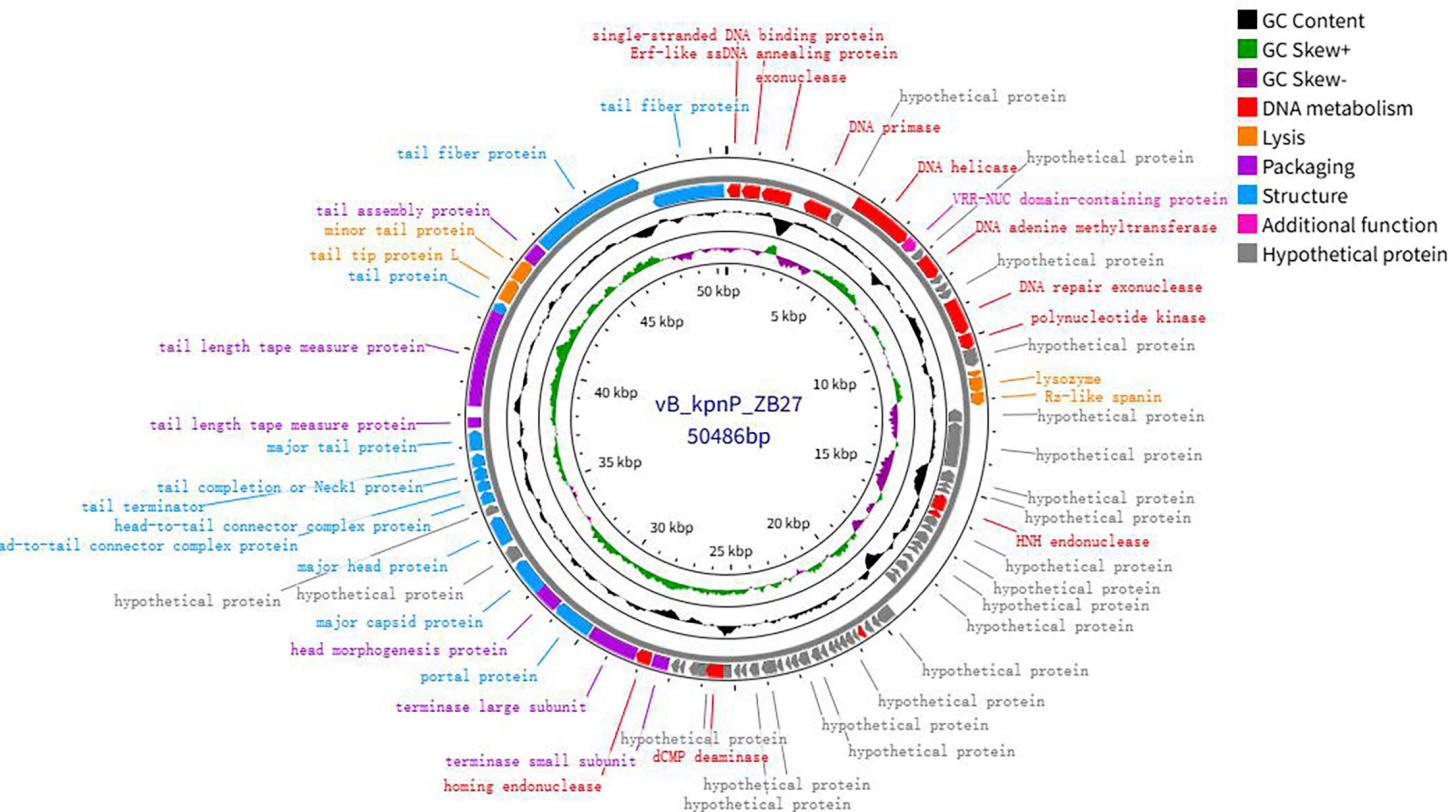
Genome map of phage ZB27. Circles display the following (from outer to inner). (i) ORFs are shown in the first and second circles. Outer circle indicates forward-strand coding, inner circle indicates reverse-strand coding. ORFs of different colors represent genes encoding different functional proteins; (ii) the third circle represents the genome’s GC content; (iii) the fourth circle shows the distribution of GC skew values across the genome; (iv) the fifth circle displays the positional coordinates of the genome sequence.

**Fig 4:**
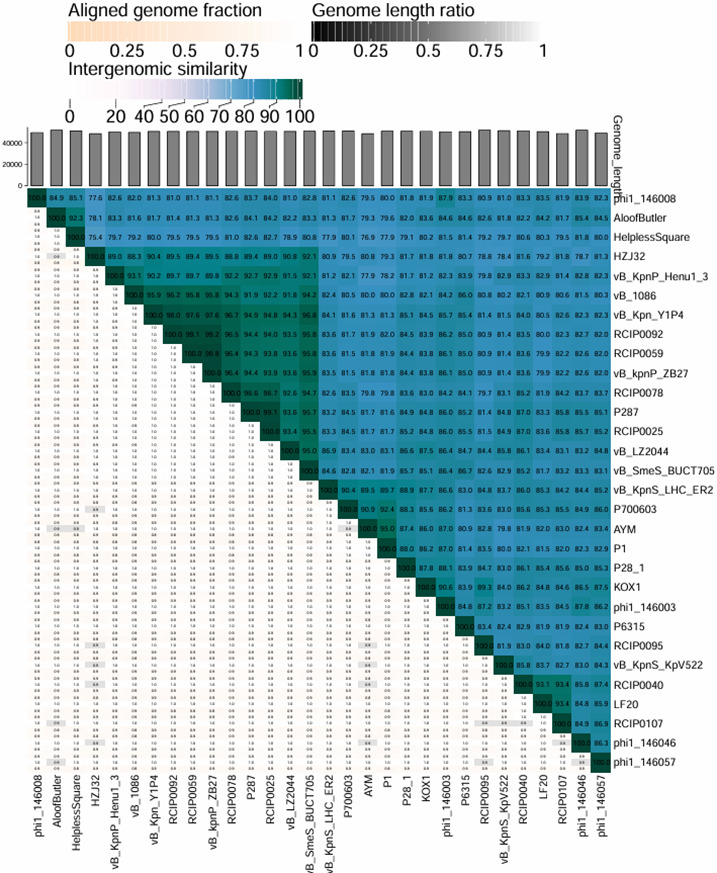
VIRIDIC heatmap. 0 (white) to 100 (dark green) represents the percentage of identity between phages.

**Fig 5:**
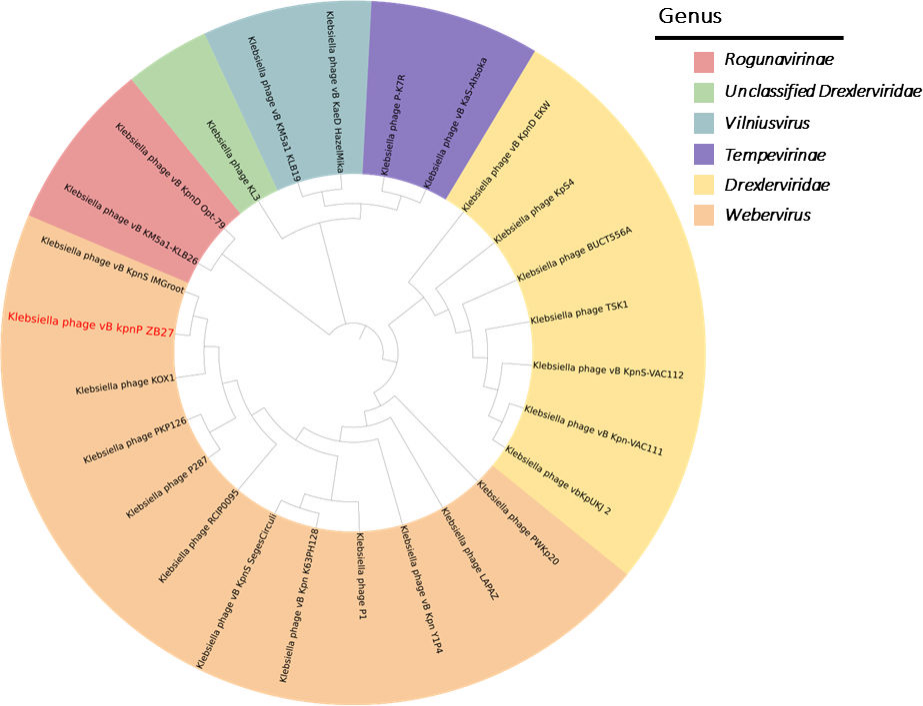
Phylogenetic tree based on the major capsid protein of phage ZB27.

**Fig 6:**
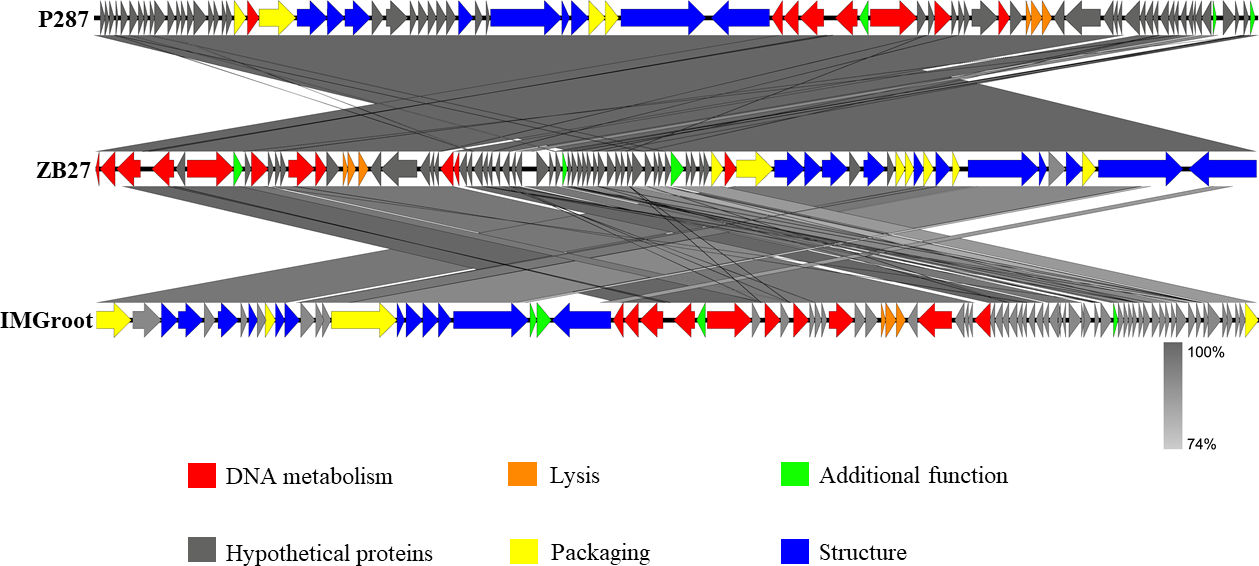
Use Easyfig for genome comparison of phage P287, ZB27, and IMGroot. Arrows in different colors represent predicted ORFs with distinct functions: putative proteins (gray), DNA metabolism (red), structural (blue), packaging (yellow), lysis (orange), and additional functions (green). Shading indicates nucleotide identity between sequences (74%−100%).

Illumina sequencing revealed the double-stranded DNA genome of phage ZB27, with a sequence length of 50,486 bp and a GC content of 50.61% (Fig. 3). BLASTn comparisons on the NCBI website indicated that phage ZB27 shares 98.28% similarity (96% coverage) with phage P287 (PP934445) and 98.20% similarity (99% coverage) with phage Y1P4 (PP663284). The VIRIDIC heatmap (Fig. 4) showed that phage ZB27 was similar to *Klebsiella* phage RCIP0078 (OR532872) (96.4%), phage P287 (PP934445) (94.4%), and phage RCIP0059 (OR532853) (99.8%). Phylogenetic analysis based on major capsid protein sequences further indicated that ZB27 belongs to the same clade as *Klebsiella* phage IMGroot (NC_049834) within the *Webeviridae* family (Fig. 5). Among the 81 open reading frames (ORFs), 44 were predicted as putative proteins, while the remaining ORFs were predicted to encode functional proteins involved in DNA metabolism, lysis, packaging, structural functions, and other functions (Fig. 3 and 6). Comparison of the genomic sequences of phage ZB27, P287, and IMGroot revealed nucleotide sequence identity consistently above 74%. The accession number of phage ZB27 in the GenBank database is PX641096.

### Effect of MOI on phage fermentation

**Fig 7:**
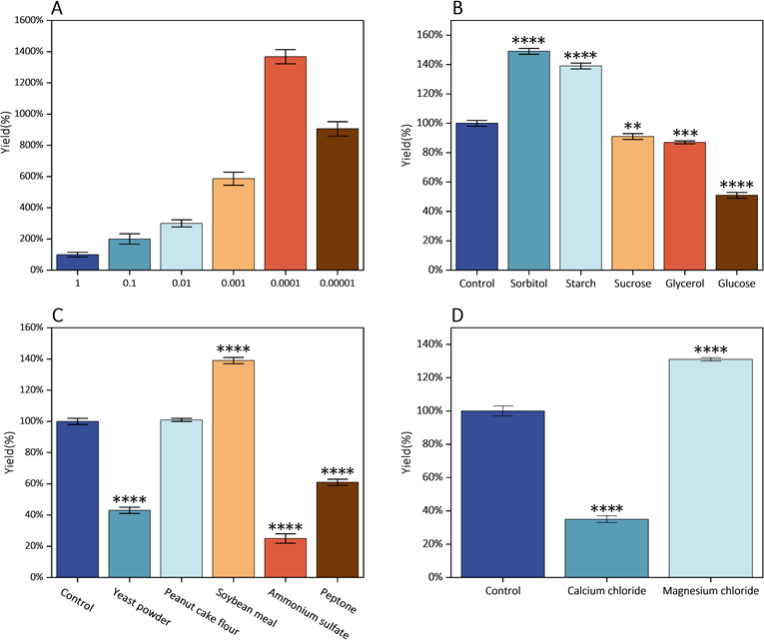
Optimization of fermentation conditions for phage ZB27. (**A**) Effect of MOI. (**B**) Effect of carbon sources. (**C**) Effect of nitrogen sources. (**D**) Effect of metal ions. The group that did not add any substances was designated as the control group. The effect of fermentation conditions on yield is shown as a percentage. Experiments were performed in triplicate. **P < 0.01, ***P < 0.001 and ****P < 0.0001.

Using a 2% ratio to inoculate the host bacteria (initial concentration: 2 × 10^7^ CFU/mL), the mixture with MOI = 1 exhibited turbidity around 4 h, indicating the start of bacterial growth. The mixtures of other groups still showed a clarified state, indicating that the phage was lysing the bacteria and replicating. At around 5 h, the mixtures with the higher and lowest MOIs (0.1, 0.01, and 0.00001) began to appear turbid. The remaining combinations showed turbidity after 6–7 h of incubation. Determining the phage titer when the mixture became turbid revealed that the highest MOI had the lowest phage yield, about 7.5 × 10^7^ PFU/mL. All other groups had phage yields greater than 10^8^ PFU/mL. The phage yield of the MOI = 0.1 group was 1.5 × 10^8^ PFU/mL and the phage yield of the MOI = 0.01 group was 2.25 × 10^8^ PFU/mL. The phage yields of the lower MOIs were higher. The phage yield for MOI = 0.001 was 4.38 × 10^8^ PFU/mL, for MOI = 0.00001 was 6.78 × 10^8^ PFU/mL, and for MOI = 0.0001, having the highest yield of more than 10^9^ PFU/mL, reaching 1.02 × 10^9^ PFU/mL (Fig. 7A). The results showed that, for phage ZB27, a low MOI can lead to a higher phage yield. MOI = 0.0001 showed the highest phage yield and was therefore used for future experiments.

### Effect of culture medium additives on phage fermentation

The host bacteria were incubated until they reached the logarithmic growth stage (OD_600_ ≈ 0.6) using NB broth. Then, they were inoculated with phage ZB27 at an MOI of 0.0001, which led to maximum phage production. Various carbon and nitrogen sources, as well as metal cations, were added to the NB broth one at a time. All growth medium additions resulted in different degrees of increase or decrease in phage production. As a carbon source, an additional 0.5% (wt/vol) of soluble starch, glucose, glycerol, sorbitol, or sucrose was added. Sorbitol and soluble starch significantly increased phage production by 49% and 39%, respectively, compared to the control without additional additives. However, glucose, glycerol, and sucrose showed varying degrees of decrease (49%, 13%, and 9%, respectively) (Fig. 7B), so sorbitol was considered the best carbon source for phage fermentation, producing 10% more phage than soluble starch. Nitrogen sources were supplemented with 0.3% (wt/vol) soybean meal, ammonium sulfate, peanut cake meal, soybean peptone, or yeast powder. Only soybean meal significantly increased phage production by 39% (Fig. 7C), while the other nitrogen sources showed insignificant increases or decreases. Therefore, soybean meal was chosen as the nitrogen source for the next experiment.

The fermentation medium was supplemented with 1 mM of calcium chloride and magnesium chloride as metal cation additives. The results showed that calcium chloride promoted phage production by 31%, while magnesium chloride had a negative effect on phage production, decreasing it by 65% (Fig. 7D). In conclusion, sorbitol, soybean meal, and calcium chloride were used as the carbon, nitrogen, and cation sources, respectively, for RSM analysis.

### RSM optimizes fermentative production of phage ZB27

After the single-factor experiment, RSM based on the BBD was used to analyze the effects of sorbitol, soybean meal, and calcium chloride on *K. pneumoniae* phage ZB27 production. Seventeen experiments were obtained from Design Expert (v.13.0.15) software, and the corresponding experimental designs and results are shown in Table 2. According to RSM analysis, the optimal code levels for sorbitol, soybean meal, and calcium chloride are: −0.44 (46.8 mg/mL), 0.99 (59.9 mg/mL), and −1 (1 mM).

**TABLE 2:** BBD for phage ZB27 production experiments

Number	Variate (coded value)			Variate (experimental value)		
	Sorbitol *X*_1_	Soybean meal *X*_2_	Calcium chloride *X*_3_	Sorbitol (mg)	Soybean meal (mg)	Calcium chloride (mmol)
1	0	1	1	250	300	5
2	0	0	0	250	200	3
3	0	0	0	250	200	3
4	0	1	−1	250	300	1
5	1	1	0	400	300	3
6	0	−1	1	250	100	5
7	0	0	0	250	200	3
8	1	0	−1	400	200	1
9	0	0	0	250	200	3
10	−1	0	1	100	200	5
11	1	0	1	400	200	5
12	1	−1	0	400	100	3
13	−1	1	0	100	300	3
14	−1	−1	0	100	100	3
15	0	0	0	250	200	3
16	−1	0	−1	100	200	1
17	0	−1	1	250	100	1

ANOVA of the model yielded the following equation, which reflects the relationship between phage production and the three factors.


$$P=291.09435+0.05998X_{1}-1.74594X_{2}-0.149713X_{3}+0.001344X_{1}X_{2}+0.44167X_{1}X_{3}-0.108334X_{2}X_{3}-0.001046X_{1}^{2}+0.0042X_{2}^{2}+0.493729X_{3}^{2}$$

**Fig 8:**
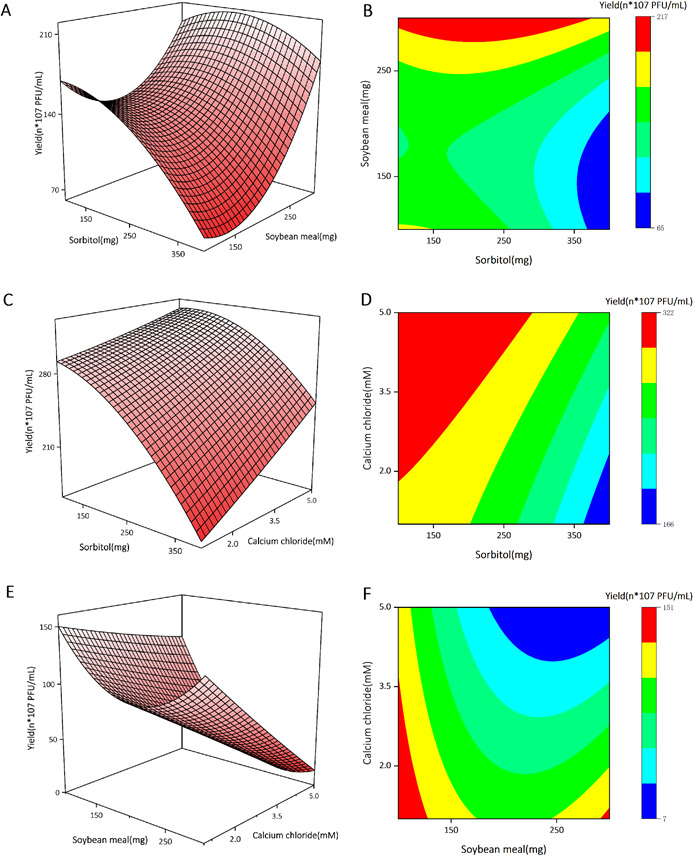
3D plots (**A, C, E**) and 2D contour plots (**B, D, F**) of the response curves of the three additives to the maximum phage yield.

The dependent variable *P* represents phage production, and the independent variables *X*_1_, *X*_2_, and *X*_3_ are the concentrations of added sorbitol, soybean meal, and calcium chloride, respectively. The experimental results of the 17 groups of production experimental designs obtained from BBD are shown in Table 3. The results of the experiment showed varying degrees of increase in the optimized yields, with the highest-yielding group (no. 4) showing an increase of 161% compared to the control group (7.43 × 10^8^ PFU/mL). Fig. 8 demonstrates a 3D plot with 2D contour plots of the response curves of the three additives to the maximum phage yield based on the experimental results

**TABLE 3:** BBD results of fermentation production experiments with phage ZB27

Number	Sorbitol (mg)	Soybean meal (mg)	Calcium chloride (mmol)	Phage production (10^9^ PFU/mL)	Projected production (10^7^ PFU/mL)
1	250	300	5	1.06 ± 0.04	120.125
2	250	200	3	1.25 ± 0.05	107.8
3	250	200	3	1.09 ± 0.07	107.8
4	250	300	1	1.94 ± 0.06	194.708
5	400	300	3	1.57 ± 0.04	142.875
6	250	100	5	1.56 ± 0.32	156.625
7	250	200	3	1.16 ± 0.04	107.8
8	400	200	1	0.64 ± 0.13	79.4167
9	250	200	3	1.06 ± 0.06	107.8
10	100	200	5	0.81 ± 0.02	66.5833
11	400	200	5	0.73 ± 0.04	74.6667
12	400	100	3	0.96 ± 0.05	95.7084
13	100	300	3	1.20 ± 0.07	120.958
14	100	100	3	1.39 ± 0.05	154.458
15	250	200	3	0.83 ± 0.06	107.8
16	100	200	1	1.25 ± 0.04	124.333
17	250	100	1	1.58 ± 0.04	144.541

### ANOVA

ANOVA was performed to assess the credibility of the model. Design Expert software (v.13.0.15) facilitated implementation. In ANOVA, high F-value and low *P*-value represent the credibility level of the model, with *P* < 0.05 indicating significance and *P* < 0.01 indicating extreme significance. In this model, high F-value (5.94) and low *P*-value (0.0142) indicate credibility (Table 4). Results of ANOVA also indicated that the coefficient of determination is 0.8842, and the F-value for lack of fit is 1.71. In summary, they all suggested that RSM can be an effective tool for optimizing *K. pneumoniae* phage ZB27 production.

**TABLE 4:** ANOVA results of fermentative production of phage ZB27

Source	DF	Mean square	*F*-value	*P*-value
Model	9	1,900.59	5.94	0.0142
*X*_1_	1	678.35	2.12	0.1887
*X*_2_	1	93.39	0.2919	0.6058
*X*_3_	1	1,953.12	6.10	0.0428
*X*_1_ *X*_2_	1	1,626.78	5.08	0.0588
*X*_1_ *X*_3_	1	702.25	2.19	0.1820
*X*_2_ *X*_3_	1	1,877.81	5.87	0.0459
*X*_1_^2^	1	2,330.19	7.28	0.0307
*X*_2_^2^	1	8,235.13	25.74	0.0014
*X*_3_^2^	1	16.42	0.0513	0.8272
Residual error	7	319.97		
Lack of fit	3	418.99	1.71	0.329
Pure error	4	245.70		
Total	6			

### Validation of optimized conditions at fermenter scale

**Fig 9:**
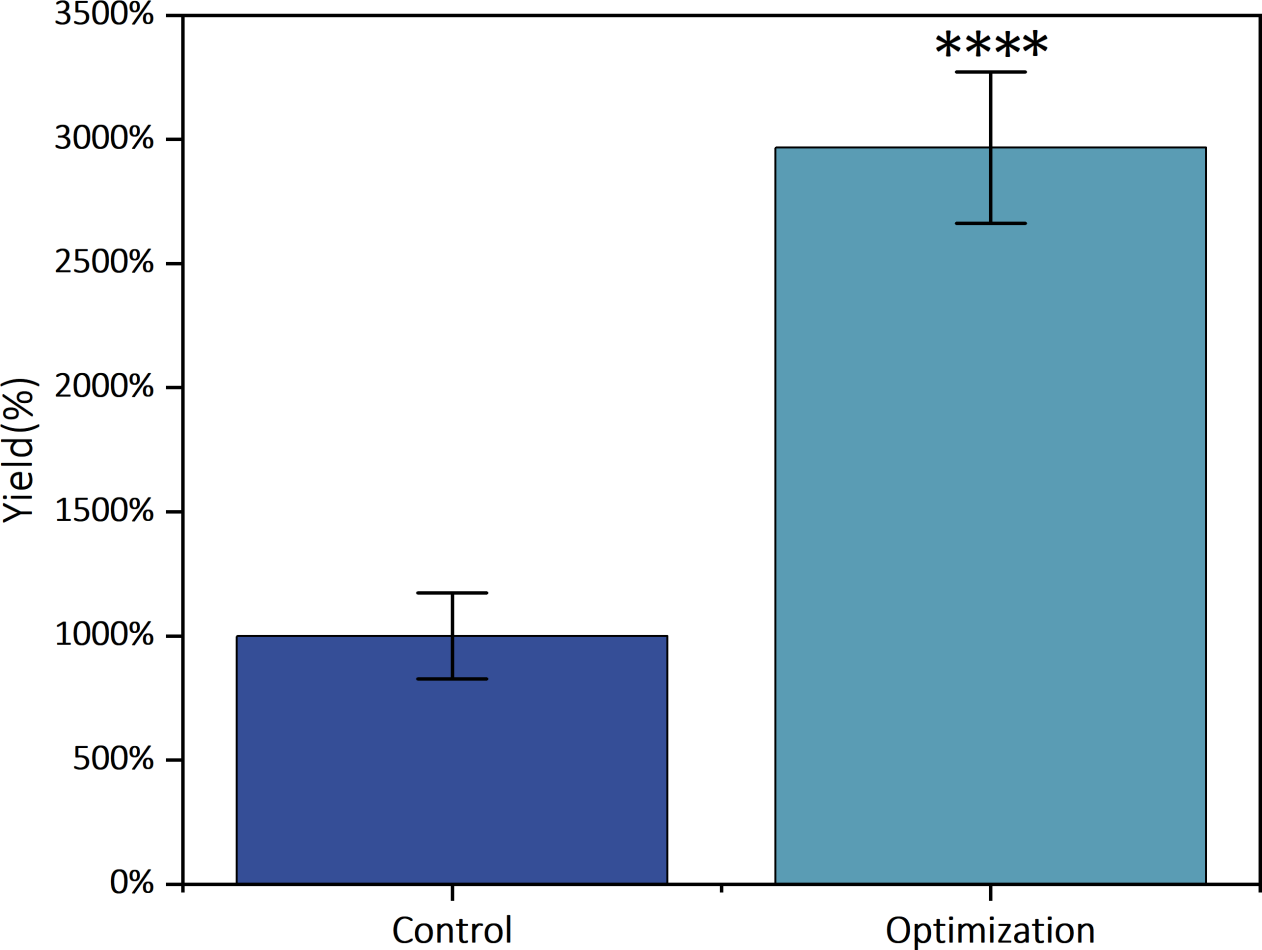
Comparison of yields after fermenter scale-up using RSM-optimized combinations ****P < 0.0001.

We employed a 50 L fully automated fermentation system to validate the RSM optimization results and scale up the production of *K. pneumoniae* phage ZB27. Fermentation conditions (37°C, 200 rpm, 8 h) matched those used in flask experiments. Phage titers were quantified post-fermentation to assess yields. The control group yielded 2.5 × 10¹³ PFU, while the RSM-optimized process achieved 7.4 × 10¹³ PFU — representing a 2.96-fold augment (Fig. 9). These results confirmed that RSM optimization significantly enhances phage production efficiency.

## DISCUSSION

In recent decades, the prevalence of highly virulent, multidrug-resistant bacteria has escalated, prompting an escalating focus on phages as a promising alternative to antibiotics for combating these pervasive threats (17, 32–36). *K. pneumoniae*, as a common pathogenic microorganism, poses a significant threat to global public health and safety due to its high pathogenicity, lethality, and drug resistance (37, 38). Consequently, a global research project has been initiated by scientists to address the challenges. This project aims to mitigate the adverse effects of *K. pneumoniae* by phage therapy (39–43). However, most of the current research has focused on the clinical applications of phages (39, 43–47). The industrial production of *K. pneumoniae* phages is currently lacking a well-established system. Prioritized efforts are urgently needed to enhance the cost-effective production of *K. pneumoniae* phages. Consequently, the exploration of practical and efficient production methods for *K. pneumoniae* phages is of significant importance. This study directly addresses this challenge. We not only established the lytic phage vB_kpnP_ZB27 as a safe and effective candidate therapeutic but, more importantly, developed a systematically optimized framework for its industrial-scale production. The initial isolation and characterization of phage ZB27 confirmed its potent therapeutic potential. Compared to other *K. pneumoniae* phages (48, 49), ZB27 possesses a broader host range and can specifically target *K. pneumoniae* strains with K1 and K2 capsules. This may be attributed to ZB27’s multiple tail fibers recognizing distinct bacterial surface receptors (50). ZB27 exhibited excellent tolerance to temperatures (20°C–50°C) and pH values (4–11); it remained active even at 60°C and at pH 3 or 12. Its specificity for clinically prevalent K1 and K2 serotypes, coupled with exceptional stability across broad temperature and pH ranges, enables reliable operation in diverse environments. Crucially, genomic analysis confirmed the absence of antibiotic resistance or pathogenicity genes, thereby affirming its safety for therapeutic use. However, these inherent biological advantages are only valuable if the phage can be produced in sufficient quantities. Therefore, the core of our work lies in optimizing the fermentation process. By adjusting phage inoculum concentration, adding nutritional supplements to the medium, and conducting experiments using RSM, we successfully enhanced phage yield through optimized fermentation conditions.

Phages cannot self-replicate without a host. Initially, phages adsorb to host surfaces (51). Higher bacterial density facilitates this adsorption by increasing collision probability between free phages and susceptible hosts (52). Previous studies have demonstrated that higher phage yields can be obtained when the host bacteria concentration is higher than the phage concentration (24, 53). The present study supports this theory. The outcome of the MOI experiment demonstrated that phage ZB27 production increased in proportion to decreasing inoculum levels, attaining its maximum yield at an inoculum level of 1/10,000 (MOI = 0.0001). However, a minimal phage inoculum of 1/100,000 led to a subsequent decline in phage yield. This phenomenon may be attributed to the restriction of initial adsorption at such low inoculum ratios, which resulted in a decrease in the subsequent production rate (19).

Supplementation of exogenous nutrients to optimize the culture medium has been a long-standing consensus in the microbial fermentation industry (54, 55). The addition of carbon and nitrogen sources to the growth medium is often essential for providing adequate nutrients. Carbon, as the fundamental element of life, is indispensable for bacterial growth, and the standard physiology of the host bacteria dictates the optimal growth of phages, just like an excellent “factory” produces good “commodities.” Although phage adsorption to the host is not terminated in a medium lacking carbon, lysis and reproduction are inhibited (56). Phage growth was promoted in an environment with a favorable carbon source; for instance, fructose increases phage pEa27 production by 432% and pEa31 production by 118% (19). Furthermore, the role of nitrogen sources in phage propagation is significant. The presence of exogenous nitrogen has been shown to facilitate the growth of host bacteria and to play a direct role in phage biosynthesis within the host (57). We observed that supplementing carbon and nitrogen sources stimulated phage production, with sorbitol (49% increase), soluble starch (39% increase), and soybean meal (39% increase) showing significant gains. It is worth noting that some of the added carbon and nitrogen sources led to a downward adjustment of phage production due to the effect of the concentration of the additives.

It is widely acknowledged that certain metal cations possess the capacity to neutralize the negative charge on the bacterial membrane surface. This neutralizing effect is considered essential for the consolidation of the binding of phage tail fibers to the membrane surface receptor (58). However, it should be noted that their effects are not universally positive. For instance, Ca^2+^ promoted the adsorption of *Lactobacillus* phage *P* 1, while Mg^2+^ showed inhibition, reflecting the variability in the effect of different ions on phage adsorption (59). Calcium ions are often thought to promote phage adsorption more than magnesium ions (17). Our study showed a 31% upregulation of phage yield with the addition of calcium ions, while magnesium ions resulted in a 65% decrease in phage yield. Compared to other gram-negative bacteria, phage adsorption is more complicated due to the presence of *K. pneumoniae* thick capsule (60). Consequently, the incorporation of metal ions to facilitate phage adsorption to *K. pneumoniae* is necessary.

The experiments to determine the optimal parameters described above were conducted on laboratory scale. However, large-scale production and application of phage requires the large bioreactors, such as large fermenters. After RSM optimization, the goal of large-scale production of *K. pneumoniae* phage ZB27 was finally achieved. We accomplished a 2.96-fold increase in production using fermentation tanks. Although our actual yield was slightly lower, our enhancement ratio compared to the unoptimized control group exceeded those reported in other literature (17, 19). However, while enhancing phage yield, the safety of phage products must not be overlooked. Centrifugation and microfiltration equipment connected to the bioreactor effectively remove residual host bacteria, while tangential flow filtration and chromatography significantly improve the purity of the phage solution. In short, the refinement of the phage production process advances phage therapy toward maturity (32).

In summary, this study has established a preliminarily optimized upstream process for the production of *K. pneumoniae* phages, providing key insights for their industrialization. With the ongoing spread of drug-resistant pathogens, there is an urgent need for effective biological control strategies targeting *K. pneumoniae*. Consequently, the industrial production of its phages holds great promise as a complementary strategy for applications in the agricultural food sector and ecological environments.

### Conclusion

This study isolated vB_kpnP_ZB27, a lytic *K. pneumoniae* phage, from sewage. The phage exhibited a specific host range, effectively lysing K1 and K2 serotypes of *K. pneumoniae*. It also demonstrated broad environmental tolerance in stability assays. Importantly, genomic analysis confirmed the absence of virulence and antibiotic resistance genes. These attributes collectively identify ZB27 as a safe and promising therapeutic candidate. Additionally, we established a production strategy for the K. *pneumoniae* phage vB_kpnP_ZB27. Higher phage yields were observed at lower inoculation concentrations, leading to the determination of an optimal MOI of 0.0001. To optimize the growth medium, sorbitol, soybean meal, and calcium chloride were supplemented. RSM optimization identified the optimal levels for these three additives. Subsequently, scaled-up production in a fermenter achieved a 2.96-fold increase in phage yield. Collectively, this strategy facilitates cost-effective, large-scale *K. pneumoniae* phage production and offers valuable insights for industrial applications.

## Data Availability

The complete phage genome sequence information referenced in the article has been submitted to GenBank and is publicly available under accession number PX641096.

## References

[1] Holmes CL, Dailey KG, Hullahalli K, Wilcox AE, Mason S, Moricz BS, Unverdorben LV, Balazs GI, Waldor MK, Bachman MA. 2025. Patterns of Klebsiella pneumoniae bacteremic dissemination from the lung. Nat Commun 16:785. doi:10.1038/s41467-025-56095-3 39824859 PMC11742683

[2] Kim JH, Jeong Y, Lee CK, Kim SB, Yoon YK, Sohn JW, Kim MJ. 2020. Characteristics of Klebsiella pneumoniae isolates from stool samples of patients with liver abscess caused by hypervirulent K. pneumoniae. J Korean Med Sci 35:e18. doi:10.3346/jkms.2020.35.e18 31920019 PMC6955435

[3] Mba IE, Mba TO, Uwazie CK, Aina FA, Kemisola AO, Uwazie IJ. 2025. New insights and perspectives on the virulence of hypervirulent Klebsiella pneumoniae. Folia Microbiol (Praha) 70:517–533. doi:10.1007/s12223-025-01261-9 40198504

[4] Wang J, Ji X, García P, Li J, Zhang L, Wang H, Wang R, He T. 2025. Evolution and transmission potential of iuc3-positive virulence plasmids in hypervirulent Klebsiella pneumoniae. Microbiol Res 299:128242. doi:10.1016/j.micres.2025.128242 40483736

[5] Dai P, Huang T, Ye X, Mi S, Zhang J, Luo X, Hu D, Zhang H. 2025. K1 Klebsiella pneumoniae is more conserved than K2 for both virulence plasmid and chromosome. BMC Genomics 26:652. doi:10.1186/s12864-025-11624-8 40640739 PMC12247478

[6] Yang Y, McNally A, Zong Z. 2025. Call for prudent use of the term hypervirulence in carbapenem-resistant Klebsiella pneumoniae. Lancet Microbe 6:101090. doi:10.1016/j.lanmic.2025.101090 39993405

[7] Furlan JPR, Savazzi EA, Stehling EG. 2020. Genomic insights into multidrug-resistant and hypervirulent Klebsiella pneumoniae co-harboring metal resistance genes in aquatic environments. Ecotoxicol Environ Saf 201:110782. doi:10.1016/j.ecoenv.2020.110782 32497817

[8] Denissen J, Reyneke B, Barnard T, Khan S, Khan W. 2023. Risk assessment of Enterococcus faecium, Klebsiella pneumoniae, and Pseudomonas aeruginosa in environmental water sources: development of surrogate models for antibiotic resistance genes. Sci Total Environ 901:166217. doi:10.1016/j.scitotenv.2023.166217 37604372

[9] Ferreira C, Luzietti L, Ribeirinho-Soares S, Nunes OC, Vaz-Moreira I, Manaia CM. 2023. Survival of clinical and environmental carbapenem-resistant Klebsiella pneumoniae ST147 in surface water. Environ Res 237:116928. doi:10.1016/j.envres.2023.116928 37607624

[10] Wyres KL, Holt KE. 2018. Klebsiella pneumoniae as a key trafficker of drug resistance genes from environmental to clinically important bacteria. Curr Opin Microbiol 45:131–139. doi:10.1016/j.mib.2018.04.004 29723841

[11] Furlan JPR, da Silva Rosa R, Ramos MS, Dos Santos LDR, Savazzi EA, Stehling EG. 2024. Emergence of carbapenem-resistant Klebsiella pneumoniae species complex from agrifood systems: detection of ST6326 co-producing KPC-2 and NDM-1. J Sci Food Agric 104:7347–7354. doi:10.1002/jsfa.13555 38651793

[12] Singh AN, Singh A, Singh SK, Nath G. 2024. Klebsiella pneumoniae infections and phage therapy. Indian J Med Microbiol 52:100736. doi:10.1016/j.ijmmb.2024.100736 39357832

[13] Yang L, Wang C, Zeng Y, Song Y, Zhang G, Wei D, Li Y, Feng J. 2024. Characterization of a novel phage against multidrug-resistant Klebsiella pneumoniae. Arch Microbiol 206:379. doi:10.1007/s00203-024-04106-0 39143367

[14] Singh A, Singh AN, Rathor N, Chaudhry R, Singh SK, Nath G. 2022. Evaluation of bacteriophage cocktail on septicemia caused by colistin-resistant Klebsiella pneumoniae in mice model. Front Pharmacol 13:778676. doi:10.3389/fphar.2022.778676 35197852 PMC8860340

[15] Fayez MS, Hakim TA, Agwa MM, Abdelmoteleb M, Aly RG, Montaser NN, Abdelsattar AS, Rezk N, El-Shibiny A. 2021. Topically applied bacteriophage to control multi-drug resistant Klebsiella pneumoniae infected wound in a rat model. Antibiotics (Basel) 10:1048. doi:10.3390/antibiotics10091048 34572629 PMC8470685

[16] Singh AN, Singh A, Nath G. 2024. Evaluation of bacteriophage cocktail on urinary tract infection caused by colistin-resistant Klebsiella pneumoniae in mice model. J Glob Antimicrob Resist 39:41–53. doi:10.1016/j.jgar.2024.07.019 39159829

[17] Kim SG, Kwon J, Giri SS, Yun S, Kim HJ, Kim SW, Kang JW, Lee SB, Jung WJ, Park SC. 2021. Strategy for mass production of lytic Staphylococcus aureus bacteriophage pSa-3: contribution of multiplicity of infection and response surface methodology. Microb Cell Fact 20:56. doi:10.1186/s12934-021-01549-8 33653327 PMC7923500

[18] Grieco SHH, Wong AYK, Dunbar WS, MacGillivray RTA, Curtis SB. 2012. Optimization of fermentation parameters in phage production using response surface methodology. J Ind Microbiol Biotechnol 39:1515–1522. doi:10.1007/s10295-012-1148-3 22714954

[19] Jo SJ, Giri SS, Lee SB, Jung WJ, Park JH, Hwang MH, Park DS, Park E, Kim SW, Jun JW, Kim SG, Roh E, Park SC. 2024. Optimization of the large-scale production for Erwinia amylovora bacteriophages. Microb Cell Fact 23:342. doi:10.1186/s12934-024-02607-7 39710718 PMC11664846

[20] Mancuso F, Shi J, Malik DJ. 2018. High throughput manufacturing of bacteriophages using continuous stirred tank bioreactors connected in series to ensure optimum host bacteria physiology for phage production. Viruses 10:537. doi:10.3390/v10100537 30275405 PMC6213498

[21] Stone E, Campbell K, Grant I, McAuliffe O. 2019. Understanding and exploiting phage-host interactions. Viruses 11:567. doi:10.3390/v11060567 31216787 PMC6630733

[22] Moebus K. 1996. Marine bacteriophage reproduction under nutrient-limited growth of host bacteria. I. Investigations with six phage-host systems. Mar Ecol Prog Ser 144:1–12. doi:10.3354/meps144001

[23] Mäkelä K, Laanto E, Sundberg LR. 2024. Determinants in the phage life cycle: the dynamic nature of ssDNA phage FLiP and host interactions under varying environmental conditions and growth phases. Environ Microbiol 26:e16670. doi:10.1111/1462-2920.16670 38952172

[24] Cheng B, Zhang D, Wang T, Chen H, Wang Y, Wang Y, Li Z, Ling N, Ye Y. 2024. Characterization of the broad-spectrum phage vB_CsaM_CBT2 and its inhibition of multidrug-resistant Cronobacter sakazakii in powered infant formula. Food Control 158:110262. doi:10.1016/j.foodcont.2023.110262

[25] Wang T, Cheng B, Jiao R, Zhang X, Zhang D, Cheng X, Ling N, Ye Y. 2024. Characterization of a novel high-efficiency cracking Burkholderia gladiolus phage vB_BglM_WTB and its application in black fungus. Int J Food Microbiol 414:110615. doi:10.1016/j.ijfoodmicro.2024.110615 38325260

[26] Cui X, Du B, Feng J, Feng Y, Fan Z, Chen J, Cui J, Gan L, Fu T, Tian Z, Zhang R, Yan C, Zhao H, Xu W, Xu Z, Yu Z, Ding Z, Li Z, Chen Y, Xue G, Yuan J. 2023. A novel phage carrying capsule depolymerase effectively relieves pneumonia caused by multidrug-resistant Klebsiella aerogenes. J Biomed Sci 30:75. doi:10.1186/s12929-023-00946-y 37653407 PMC10470133

[27] Moraru C, Varsani A, Kropinski AM. 2020. VIRIDIC-a novel tool to calculate the intergenomic similarities of prokaryote-infecting viruses. Viruses 12:1268. doi:10.3390/v12111268 33172115 PMC7694805

[28] Zimmermann L, Stephens A, Nam SZ, Rau D, Kübler J, Lozajic M, Gabler F, Söding J, Lupas AN, Alva VA. 2018. A completely reimplemented MPI bioinformatics toolkit with a new HHpred server at its core. J Mol Biol 430:2237–2243. doi:10.1016/j.jmb.2017.12.007 29258817

[29] Stothard P, Grant JR, Van Domselaar G. 2019. Visualizing and comparing circular genomes using the CGView family of tools. Brief Bioinformatics 20:1576–1582. doi:10.1093/bib/bbx081 28968859 PMC6781573

[30] Sullivan MJ, Petty NK, Beatson SA. 2011. Easyfig: a genome comparison visualizer. Bioinformatics 27:1009–1010. doi:10.1093/bioinformatics/btr039 21278367 PMC3065679

[31] Nishimura Y, Yoshida T, Kuronishi M, Uehara H, Ogata H, Goto S. 2017. ViPTree: the viral proteomic tree server. Bioinformatics 33:2379–2380. doi:10.1093/bioinformatics/btx157 28379287

[32] Skurnik M, Alkalay-Oren S, Boon M, Clokie M, Sicheritz-Pontén T, Dąbrowska K, Hatfull GF, Hazan R, Jalasvuori M, Kiljunen S, Lavigne R, Malik DJ, Nir-Paz R, Pirnay J-P. 2025. Phage therapy. Nat Rev Methods Primers 5:9. doi:10.1038/s43586-024-00377-5

[33] Nobrega FL, Costa AR, Kluskens LD, Azeredo J. 2015. Revisiting phage therapy: new applications for old resources. Trends Microbiol 23:185–191. doi:10.1016/j.tim.2015.01.006 25708933

[34] Almeida G de F, Sundberg L-R. 2020. The forgotten tale of Brazilian phage therapy. Lancet Infect Dis 20:e90–e101. doi:10.1016/S1473-3099(20)30060-8 32213334

[35] Monteiro R, Pires DP, Costa AR, Azeredo J. 2019. Phage therapy: going temperate? Trends Microbiol 27:368–378. doi:10.1016/j.tim.2018.10.008 30466900

[36] Kushwaha SO, Sahu SK, Yadav VK, Rathod MC, Patel D, Sahoo DK, Patel A. 2024. Bacteriophages as a potential substitute for antibiotics: a comprehensive review. Cell Biochem Funct 42:e4022. doi:10.1002/cbf.4022 38655589

[37] Das M. 2024. Global update on hypervirulent Klebsiella pneumoniae. Lancet Infect Dis 24:e621. doi:10.1016/S1473-3099(24)00610-8 39341224

[38] Capone A, Giannella M, Fortini D, Giordano A, Meledandri M, Ballardini M, Venditti M, Bordi E, Capozzi D, Balice MP, Tarasi A, Parisi G, Lappa A, Carattoli A, Petrosillo N. 2013. High rate of colistin resistance among patients with carbapenem-resistant Klebsiella pneumoniae infection accounts for an excess of mortality. Clin Microbiol Infect 19:E23–E30. doi:10.1111/1469-0691.12070 23137235

[39] Le Bris J, Chen N, Supandy A, Rendueles O, Van Tyne D. 2025. Phage therapy for Klebsiella pneumoniae: Understanding bacteria-phage interactions for therapeutic innovations. PLoS Pathog 21:e1012971. doi:10.1371/journal.ppat.1012971 40198880 PMC11978313

[40] Lin TL, Yang FL, Ren CT, Pan YJ, Liao KS, Tu IF, Chang YP, Cheng YY, Wu CY, Wu SH, Wang JT. 2022. Development of Klebsiella pneumoniae capsule polysaccharide-conjugated vaccine candidates using phage depolymerases. Front Immunol 13:843183. doi:10.3389/fimmu.2022.843183 35386691 PMC8978995

[41] Martins W, Li M, Sands K, Lenzi MH, Portal E, Mathias J, Dantas PP, Migliavacca R, Hunter JR, Medeiros EA, Gales AC, Toleman MA. 2022. Effective phage cocktail to combat the rising incidence of extensively drug-resistant Klebsiella pneumoniae sequence type 16. Emerg Microbes Infect 11:1015–1023. doi:10.1080/22221751.2022.2051752 35259067 PMC9004492

[42] Wang C, Wang S, Jing S, Zeng Y, Yang L, Mu Y, Ding Z, Song Y, Sun Y, Zhang G, Wei D, Li M, Ma Y, Zhou H, Wu L, Feng J. 2024. Data‐driven engineering of phages with tunable capsule tropism for Klebsiella pneumoniae. Adv Sci (Weinh) 11. doi:10.1002/advs.202309972 PMC11434222 38937990

[43] Eskenazi A, Lood C, Wubbolts J, Hites M, Balarjishvili N, Leshkasheli L, Askilashvili L, Kvachadze L, van Noort V, Wagemans J, Jayankura M, Chanishvili N, de Boer M, Nibbering P, Kutateladze M, Lavigne R, Merabishvili M, Pirnay J-P. 2022. Combination of pre-adapted bacteriophage therapy and antibiotics for treatment of fracture-related infection due to pandrug-resistant Klebsiella pneumoniae. Nat Commun 13:302. doi:10.1038/s41467-021-27656-z 35042848 PMC8766457

[44] Abbas S, Kanwar R, Ullah K, Kanwal R, Tajamal M, Aslam MA, Ahmad A, Qadeer A, Huang HY, Chen CC. 2025. Bacteriophage therapy: a possible alternative therapy against antibiotic-resistant strains of Klebsiella pneumoniae Front Microbiol 16:1443430. doi:10.3389/fmicb.2025.1443430 40231234 PMC11994585

[45] Ichikawa M, Nakamoto N, Kredo-Russo S, Weinstock E, Weiner IN, Khabra E, Ben-Ishai N, Inbar D, Kowalsman N, Mordoch R, et al.. 2023. Bacteriophage therapy against pathological Klebsiella pneumoniae ameliorates the course of primary sclerosing cholangitis. Nat Commun 14:3261. doi:10.1038/s41467-023-39029-9 37277351 PMC10241881

[46] Gholizadeh O, Ghaleh HEG, Tat M, Ranjbar R, Dorostkar R. 2024. The potential use of bacteriophages as antibacterial agents against Klebsiella pneumoniae. Virol J 21:191. doi:10.1186/s12985-024-02450-7 39160541 PMC11334591

[47] Xing J, Han R, Zhao J, Zhang Y, Zhang M, Zhang Y, Zhang H, Nang SC, Zhai Y, Yuan L, Wang S, Wu H. 2025. Revisiting therapeutic options against resistant Klebsiella pneumoniae infection: phage therapy is key. Microbiol Res 293:128083. doi:10.1016/j.micres.2025.128083 39904002

[48] Yan T, Wang Q, Ma C, Teng X, Gong Z, Chu W, Zhou Q, Liu Z. 2025. Phage vB_Kpn_HF0522: isolation, characterization, and therapeutic potential in combatting K1 Klebsiella pneumoniae infections. Infect Drug Resist 18:803–818. doi:10.2147/IDR.S501921 39958984 PMC11827489

[49] Chen C, Tao Z, Li T, Chen H, Zhao Y, Sun X. 2023. Isolation and characterization of novel bacteriophage vB_KpP_HS106 for Klebsiella pneumonia K2 and applications in foods. Front Microbiol 14:1227147. doi:10.3389/fmicb.2023.1227147 37655345 PMC10466807

[50] Yin M, Cao L, Fu Y, Lu Y, Yan Y, Qian L, Xiang L, Zhou T, Chen H, Li Y, Zhang L. 2025. A novel tail fiber protein triggers phage DNA ejection by recognizing lipopolysaccharides of K54 hypervirulent Klebsiella pneumoniae Microbiol Spectr 13:e0217125. doi:10.1128/spectrum.02171-25 41212188 PMC12671077

[51] Ge H, Hu M, Zhao G, Du Y, Xu N, Chen X, Jiao X. 2020. The “fighting wisdom and bravery” of tailed phage and host in the process of adsorption. Microbiol Res 230:126344. doi:10.1016/j.micres.2019.126344 31561173

[52] Abedon ST. 2016. Phage therapy dosing: the problem(s) with multiplicity of infection (MOI). Bacteriophage 6:e1220348. doi:10.1080/21597081.2016.1220348 27738558 PMC5056779

[53] Feng J, Cui X, Du B, Chen J, Xue G, Gan L, Feng Y, Fan Z, Ke Y, Cui J, Fu T, Zhao H, Yan C, Xu Z, Yang Y, Yu Z, Huang L, Zhao S, Tian Z, Ding Z, Chen Y, Li Z, Yuan J. 2024. Characterization of novel phage pK3-24 targeting multidrug-resistant Klebsiella pneumoniae and its therapeutic efficacy in Galleria mellonella larvae. Virus Res 350:199481. doi:10.1016/j.virusres.2024.199481 39395674 PMC11533715

[54] Zhang J, Yao T, Gong W, Gao Y, Zhao G. 2023. Additive screening and formula optimization of microbial inhibitor having disease prevention and growth promotion effects on Avena sativa. Front Microbiol 14:1208591. doi:10.3389/fmicb.2023.1208591 37547695 PMC10397394

[55] Tsouko E, Kachrimanidou V, Dos Santos AF, do Nascimento Vitorino Lima ME, Papanikolaou S, de Castro AM, Freire DMG, Koutinas AA. 2017. Valorization of by-products from palm oil mills for the production of generic fermentation media for microbial oil synthesis. Appl Biochem Biotechnol 181:1241–1256. doi:10.1007/s12010-016-2281-7 27787766

[56] Briggiler Marcó M, Reinheimer J, Quiberoni A. 2015. Phage adsorption and lytic propagation in Lactobacillus plantarum: could host cell starvation affect them? BMC Microbiol 15:273. doi:10.1186/s12866-015-0607-1 26627203 PMC4667525

[57] Waldbauer JR, Coleman ML, Rizzo AI, Campbell KL, Lotus J, Zhang L. 2019. Nitrogen sourcing during viral infection of marine cyanobacteria. Proc Natl Acad Sci USA 116:15590–15595. doi:10.1073/pnas.1901856116 31308237 PMC6681717

[58] Carlson HK, Piya D, Moore ML, Magar RT, Elisabeth NH, Deutschbauer AM, Arkin AP, Mutalik VK. 2023. Geochemical constraints on bacteriophage infectivity in terrestrial environments. ISME Commun 3:78. doi:10.1038/s43705-023-00297-7 37596312 PMC10439110

[59] Chen X, Xi Y, Zhang H, Wang Z, Fan M, Liu Y, Wu W. 2016. Characterization and adsorption of Lactobacillus virulent phage P1. J Dairy Sci 99:6995–7001. doi:10.3168/jds.2016-11332 27372579

[60] Li P, Ma W, Cheng J, Zhan C, Lu H, Shen J, Zhou X. 2025. Phages adapt to recognize an O-antigen polysaccharide site by mutating the “backup” tail protein ORF59, enabling reinfection of phage-resistant Klebsiella pneumoniae. Emerg Microbes Infect 14:2455592. doi:10.1080/22221751.2025.2455592 39817558 PMC11795761

